# Spiritual Care as Part of an Interprofessional Model for Debriefing on an Oncology Unit

**DOI:** 10.1177/15423050211021387

**Published:** 2021-11-12

**Authors:** Kalli (Kalliopi) Stilos, Bill Ford, Anita Chakraborty, Danielle Takahashi

**Affiliations:** Sunnybrook Health Sciences Centre, Canada; University of Toronto, Canada; Sunnybrook Health Sciences Centre, Canada; Sunnybrook Health Sciences Centre, Canada; University of Toronto, Canada; Sunnybrook Health Sciences Centre, Canada

**Keywords:** Spiritual care, debrief sessions, self-care, oncology, burnout

## Abstract

Health care clinicians who care for seriously ill and dying patients have been known to be at higher risk for burnout and moral distress. When not well supported in their workplace, clinicians may suffer additional adverse outcomes to their overall wellbeing. Self-care is one way to help mitigate these adverse outcomes. The literature has described formalized debriefing not only as a self-care strategy but an intervention to promote healthy team development. The focus of this paper will showcase how social work and spiritual care practitioners in our institution worked collaboratively to support an inpatient oncology unit to address issues related to burnout by providing staff with monthly debriefing sessions intended to enhance self-care and wellness in the workplace.

## Introduction

In 2016 Sunnybrook Health Sciences launched the “Quality Living and Dying Initiative” (QLDI) as a corporate commitment to ensuring high quality palliative and end of life care to dying patients and their families (Stilos et al., 2016). In addition to prioritizing patient care, the QLDI acknowledges that health care providers working with dying patients must also demonstrate awareness of their own emotional status including the impact of caring for patients with complex needs at end of life (Freeman, 2013, p. 151). It is widely reported that health care providers who care for seriously ill patients are at higher risk for burnout, moral distress, and compassion fatigue (Sanchez-Reilly et al., 2013, p. 75). Self-care is one way to help mitigate these adverse outcomes.

Self-care is a spectrum of knowledge, skills, and attitudes including self-reflection and self-awareness, identification and prevention of burnout and compassion fatigue, setting appropriate boundaries, and designated time for grief and bereavement (Sanchez-Reilly et al., 2013). Self-care is practiced via various modalities such as debriefing, communication, meditation, and other methods to promote emotional health (Freeman, 2013, p. 151). Promoting self-care and resilience are integral to clinicians who routinely witness distress that is inherent in populations facing critical illness and death.

Improving one’s wellness involves implementation of self-care strategies, including attention to both personal and professional self-care (Chittenden & Ritchie, 2011). Personal self-care includes time with our families, our community and support networks, and attending to spirituality as defined by the individual. Elements may include maintaining a healthy lifestyle including regular exercise, vacations, hobbies, and work-life harmony (Bowman, 2007; Dyrbye et al., 2011; Shanafelt et al., 2005). Strategies such as practicing mindfulness and meditation, reflective writing or journaling, reciting mantras and pursuing spiritual development have also been described (Cohen-Katz et al., 2004; Puchalski & Guenther, 2012). Routine incorporation of self-care strategies has many positive outcomes such as minimizing burnout, compassion fatigue, and moral distress (Sanchez-Reilly et al., 2013). For health care providers there is positive potential for job engagement, compassion, satisfaction and resilience (Sanchez-Reilly et al., 2013). However, it has been noted that increased clinical wellbeing and resilience must encompass more than just personal self-care efforts. Corporate strategies may include peer support groups, interventions focused on addressing organizational culture, practices, and operating systems (Hunt, 2020). Clinicians’ wellbeing and resilience may suffer in settings that lack wellness practices such as staff appreciation, reflection, and a sense of community (Hunt, 2020). It has been noted that the aftermath of distressing events can go concealed for years and on occasion clinicians have noted them manifest through somatic, psychological and behavioral changes (Bard & Bursztajn, 2020).

Self-care is an essential component of professional development in palliative care (Mills et al., 2018). The literature describes both individual and group-oriented strategies for self-care for palliative care clinicians (Podgurski et al.2019). Orellan-Rios et al. (2018) describes “on the job” mindfulness practices incorporated into the weekly schedule of a palliative care team. The authors noted specific improvements in awareness and resilience of team members upon completion of the intervention, as well as benefits to team communication and conflict management. A study exploring moral distress in chaplains found that those working in palliative care reported higher rates of moral distress and burnout (White et al., 2019). Those who engaged in routine self-care demonstrated lower rates of distress and one-third of chaplains highlighted the importance of engaging in weekly formal debriefings sessions with interprofessional colleagues.

Corporate leadership plays an important role by prioritizing and affording routine opportunities for self-care. At our institution, the department of Organizational Development routinely circulates resources and practical tools to enhance staff wellness, such as information on peer support groups, mindfulness groups, one-on-one peer support sessions, and other specific resources tailored to the individual’s needs. Providing staff with various self-care options may encourage individuals to adopt strategies according to personal preference or learning styles. Our institution is the first acute care hospital in our region to provide “Schwartz Rounds”. Strong leadership demonstrated by the spiritual care team was instrumental in bringing the initiative to fruition at our hospital. Spiritual Care provides ongoing coordination of the rounds and facilitates group discussions after each presentation. The rounds are made available to all employees of the hospital and cover a range of topics that are relevant to an interprofessional health care teams. To date the rounds have been very well attended. Providing a safe forum for discussion amongst staff. Schwartz rounds allow “… healthcare providers a regularly scheduled time during their fast-paced work lives to openly and honestly discuss the social and emotional issues they face in caring for patients and families” (The Schwartz Centre for Compassionate Health Care, 2021). This creates the space for staff to be “… better able to make personal connections with patients and colleagues when they have greater insight into their own responses and feelings” (The Schwartz Centre for Compassionate Health Care 2021). Schwartz rounds have continued at our institution via an electronic platform during the COVID-19 global pandemic, thereby providing staff with much needed opportunities for ongoing self-care.

Formalized debriefing has been described in the literature as both a self-care strategy and as a strategy to promote healthy team development (Mills et al.. 2018). Optimal team functioning is especially important in the context of interprofessional palliative care teams which routinely care for physically and emotionally complex patients. A recent study examined the effects of reflective debriefing sessions on moral distress of ICU nurses (Browning & Cruz 2018). These sessions included both reflective and educational components and were facilitated by social work colleagues. The majority of participants in the pilot project reported support for ongoing monthly debriefing sessions.

The focus of this paper is to showcase how social work and spiritual care practitioners worked collaboratively to support an inpatient oncology unit by providing staff with monthly debriefing sessions intended to enhance self-care and wellness in the workplace.

## Ethics Review

Institutional ethics review was not required for this project as determined by the Sunnybrook Health Sciences Ethics Review Self-Assessment Tool (ER-SAT) and confirmed with the Ethics Office.

## Debriefing Process

The process of debriefing entails a discussion between health care providers and an exchange of information-sharing and event-processing. Group participants become informants to each other about a situation or event that they experienced as a group. The facilitator can be a therapist, counselor, or professional peer who helps the group participants to process the information being shared.

Although there was no formalized debriefing education available to the facilitators in our institution, both social workers and chaplains through their professional requirements are trained in psychosocial assessment, group facilitation, and conflict management. The social worker and the spiritual care practitioner for the inpatient oncology unit each have the professional skills to guide the debriefing process, which is focused on exploring wellness and distress amongst staff. In addition, they have knowledge about organizational and community supports which may be relevant to support participants. These facilitators also possessed the knowledge and skills to assess which staff may benefit from additional individual counseling, resources, and support, as recommended in the literature (Lim et al., 2000).

## Description of the Debrief

The monthly debriefing sessions occurred on an inpatient oncology unit that serves an incredibly complex patient population with a consistently high standard of care provided by staff. Sessions were open to the entire interprofessional team including nursing, physicians, pharmacists, allied health and the unit manager. A disclaimer was noted at the beginning of the debriefs about confidentially and about the voluntary nature of the debriefs. A record of attendance was not taken as they are informal and voluntary and not meant for crediting healthcare staff for attendance.

The purpose of the monthly debrief was to focus on staff self-care and wellbeing. Along with a pre-determined topic of discussion for each debrief, there was coffee, tea and light refreshments which often enticed already busy staff to pause, take a break, and allow some time for themselves with their colleagues.

The topics or themes of each debrief varied each month depending on the perceived needs of the team at the time. Staff discussion and participation were elicited through team building exercises, games, discussing articles on current developments in self-care, and encouraging team members to discuss their strategies for maintaining resilience. Where it was applicable literature would be referenced and provided to the group by the facilitators in collaboration with the appropriate health experts on the subject matter. Some examples of topics included meditation, eating habits to nourish mind and body, formulating a gratitude practice, cultivating personal happiness, as well as personal and professional goal setting. When caseloads or hospital occupancy were particularly high, staff may have felt overworked and less connected to their patients or work. In these instances, the topics of discussion focused on exploring sources of meaning and purpose in staff’s professional and personal lives. When there were new staff members or students, the sessions would shift to team building exercises intended to facilitate closer connections between newer and senior staff on the unit.

Although a structured topic or theme was useful to guide conversation during the debrief, it was equally important for the facilitators to remain flexible depending on the emotional needs of individual staff. For example, if tensions were running particularly high and staff opted not to participate in a structured discussion, facilitators would initiate short games to help break the tension. Another strategy was a “build-your-own” sundae or cupcake station as a way to offer staff a moment to simply sit and rest. Often the simple act of recognizing hard work with a cup of coffee or snack was all that was needed to demonstrate compassion and understanding.

While routine debriefing sessions were facilitated on a monthly basis, ad hoc debriefs also occurred during particularly emotional or challenging times. These usually occurred the same day or shortly after a critical event. Examples include the unexpected termination of a team member and catastrophic bleeding in a patient at end of life. One particular case revolved around the death of a young patient to whom the whole team was extremely connected. The patient had been admitted to the unit at the age of 18 after his initial diagnosis, and subsequently had multiple admissions over the trajectory of his illness. As the patient approached end of life during his final admission, both the patient’s family and team felt that the inpatient oncology unit was “home” to the patient. It was decided that transfer to a palliative care unit would have been emotionally detrimental to all involved. Following the patient’s death, the entire interprofessional team gathered to debrief. Team members reflected on their experiences of caring for the patient and family. They reflected on the patient’s death, and their own disenfranchised grief while simultaneously caring for other patients on the unit. Various coping strategies were explored. Even the patient’s primary oncologist, felt they had a safe place to weep openly and express their grief with “staff who understood the gravity of the situation”. The entire team was provided with a safe and compassionate environment in which to express and share grief, to pause and pay respect to the patient, and to explore the impact of caring for this patient throughout the healthcare journey.

Facilitating debrief after critical events was essential to provide staff with a safe forum in which to reflect upon the varying degrees of distress or emotion they experienced. It encouraged them to express the impact of patient care on their personal and professional lives. Sharing in a group setting helped diminish feelings of isolation experienced by care providers and explicitly acknowledged the impacts on mental health and resilience. The sessions helped to dispel the myth that patients are treated as “just another number” in a busy hospital setting. Facilitators acknowledged that providing high quality clinical care often affects the well-being of staff in their daily work. The recognition allowed staff to continue caring for patients in an authentic and engaged way, and with a renewed sense of the worth and meaning of their continued contributions to patient care.

Staff who self-identified they needed additional supports during the debriefing sessions were encouraged to seek approved organizational sources of support through the Occupational Health and Safety department, the Wellbeing Sunnybrook Team, and the Employee Assistance Program: Homewood Health program. These resources are free of charge to all staff and the facilitators always had the contact information readily available in written form or could provide them electronically if needed.

## Feedback to Facilitators

Although debriefs were not formally evaluated, staff often expressed gratitude and positive feedback after each debrief, noting how “it was nice to have a few moments for ourselves”. Staff valued the opportunity to connect with colleagues in a meaningful way in order to support a cohesive and high performing team. For those who participated in the debriefing sessions regularly, this often translated to closer relationships amongst team members. Facilitators noted that staff were more likely to engage in informal check-ins amongst themselves, focusing on workload, patient care, and coping with stressors outside of work. It should be noted that each of the facilitators were valued team members, which contributed to creating a safe space for staff to express emotions related to both successes and challenges. Staff regarded the monthly debrief as an opportunity to express themselves and how they were coping with the demands of the work and patient population. Staff were able to return to their work day with a sense of community amidst the various pressure and stressors they felt, and reported having more strategies to manage these stressors. Our experience aligns with other studies (Hunt, 2020) which note health professionals appreciate the opportunity for “space to breathe”, self-reflection, mindfulness, and self-care while developing professional robustness and sense of value and team identity.

## Limitations

The debriefing sessions examined were limited to only one medical oncology unit in our organization and therefore may not be applicable to non-oncology settings. Participants did not formally evaluate the impact of the debriefing sessions. In some instances conflicts of interest arose if the facilitator was involved in the critical event being explored. In such cases there was the opportunity for a clinical ethicist to facilitate the debriefing session. In addition, the COVID pandemic impacted the social worker and spiritual care caseloads and workload as their priority was to support patients’ connections to family and provide social supports. During the strict lockdown periods group debriefs were canceled as COVID guidelines discouraged congregating in large groups creating another barrier to healthcare workers coming together.

## Conclusion

Healthcare workers who provide care to patients with complex needs are at higher risk for moral distress and burnout. As such, organizations must actively support staff wellness via various modalities. Our experience suggests that monthly facilitated staff debriefing sessions are a sustainable option to support clinicians grappling with issues of burnout, compassion fatigue, and moral distress.
